# The Optimization Design of Macrophage Membrane Camouflaging Liposomes for Alleviating Ischemic Stroke Injury through Intranasal Delivery

**DOI:** 10.3390/ijms25052927

**Published:** 2024-03-02

**Authors:** Tianshu Liu, Yan Wang, Mengfan Zhang, Jin Zhang, Naijin Kang, Linlin Zheng, Zhiying Ding

**Affiliations:** School of Pharmaceutical Sciences, Jilin University, Changchun 130021, China; tsliu21@mails.jlu.edu.cn (T.L.); shenmary@163.com (Y.W.); zmf21@mails.jlu.edu.cn (M.Z.); zjin21@mails.jlu.edu.cn (J.Z.); kangnj22@mails.jlu.edu.cn (N.K.); llzheng22@mails.jlu.edu.cn (L.Z.)

**Keywords:** biomimetic liposome, combination therapy, ischemic stroke, intranasal administration, network pharmacology

## Abstract

Ischemic stroke is associated with a high mortality rate, and effective treatment strategies are currently lacking. In this study, we aimed to develop a novel nano delivery system to treat ischemic stroke via intranasal administration. A three-factor Box–Behnken experimental design was used to optimize the formulation of liposomes co-loaded with Panax notoginseng saponins (PNSs) and Ginsenoside Rg3 (Rg3) (Lip-Rg3/PNS). Macrophage membranes were coated onto the surface of the optimized liposomes to target the ischemic site of the brain. The double-loaded liposomes disguised by macrophage membranes (MM-Lip-Rg3/PNS) were spherical, in a “shell–core” structure, with encapsulation rates of 81.41% (PNS) and 93.81% (Rg3), and showed good stability. In vitro, MM-Lip-Rg3/PNS was taken up by brain endothelial cells via the clathrin-dependent endocytosis and micropinocytosis pathways. Network pharmacology experiments predicted that MM-Lip-Rg3/PNS could regulate multiple signaling pathways and treat ischemic stroke by reducing apoptosis and inflammatory responses. After 14 days of treatment with MM-Lip-Rg3/PNS, the survival rate, weight, and neurological score of middle cerebral artery occlusion (MCAO) rats significantly improved. The hematoxylin and eosin (H&E) and TUNEL staining results showed that MM-Lip-Rg3/PNS can reduce neuronal apoptosis and inflammatory cell infiltration and protect the ischemic brain. In vivo biological experiments have shown that free Rg3, PNS, and MM-Lip-Rg3/PNS can alleviate inflammation and apoptosis, especially MM-Lip-Rg3/PNS, indicating that biomimetic liposomes can improve the therapeutic effects of drugs. Overall, MM-Lip-Rg3/PNS is a potential biomimetic nano targeted formulation for ischemic stroke therapy.

## 1. Introduction

Ischemic stroke is a severe cardiovascular disease, with a high incidence rate worldwide, that seriously endangers human health [1,2]. The panax notoginseng saponins (PNSs) are extracted from the roots of *Panax notoginseng (Burk.) F. H. Chen*, and their main active ingredients include five components: ginsenoside Rg1 (Rg1), ginsenoside Rb1 (Rb1), ginsenoside Rd (Rd), ginsenoside Re (Re), and notoginsenoside R1 (R1) [3,4]. Extensive scientific research has shown that PNS has pharmacological activity, such as anti-inflammatory effects, angiogenesis promotion, thrombosis inhibition, and antioxidant properties [5,6,7]. It can reduce brain damage during ischemia and hypoxia and has significant therapeutic effects on ischemic stroke [8,9]. Ginsenoside Rg3 (Rg3) originates from ginseng and is an active ingredient [10]. It has anti-inflammatory and antioxidant effects, making it important for the treatment of ischemic stroke [11,12]. The two drugs have a common anti-ischemic effect and can produce therapeutic effects on multiple targets in ischemic stroke when used in a single formulation.

Despite their usefulness, the inherent characteristics of PNS (water solubility and high molecular weight, difficulty crossing the blood-brain barrier) and Rg3 (extremely low solubility, difficulty crossing the blood–brain barrier, and poor bioavailability) limit the treatment of ischemic stroke [13,14,15]. Nanocarriers such as nanoparticles [16], nano emulsions [17], liposomes [18], and micelles [19], which improve drug bioavailability and increase drug stability and solubility [20,21], have attracted much attention in recent years [22]. Among them, liposomes were the first nanomedicines approved by the FDA [23]. Liposomes exhibit unique advantages as nanocarriers. Liposomes are composed of non-toxic phospholipids and cholesterol and have a structure similar to that of a biofilm [24,25]. Therefore, compared to other nanocarriers, they have better biocompatibility and lower toxicity [26,27]. The bilayer structure of the liposomes allows them to simultaneously encapsulate two drugs with different properties: hydrophilic and hydrophobic [28,29]. Therefore, liposomes are the most suitable nanocarriers for co-loading two different soluble drugs to achieve anti-cerebral ischemic drug delivery [30].

Although ordinary liposomes can achieve effective drug loading, they lack active targeting capabilities. To ensure maximum efficacy and minimal toxic side effects, it is crucial to construct a nano delivery system with a specific targeting ability towards the lesion [31]. Cell membrane-coated nanomaterials have become a new and promising nanotechnology [32,33]. This biomimetic nano delivery system’s uniqueness lies in its ability to utilize affinity ligands on the cell membrane to achieve targeted therapy for diseases, thereby improving its therapeutic effect [34,35]. For example, macrophage membrane-coated liposomes can play an important role in targeted drug delivery; these stealth liposomes allow liposomal cloaking into macrophage membranes and can protect the liposomes from phagocytic uptake by the immune cells, whereas the surface-wrapped macrophage membranes allow for the recognition of antigens and target inflamed tissues [36]. These biomimetic nanocarriers have a “shell–core” structure, with different nanoparticles inside and a cell membrane on the outer layer [37,38]. Common cell membranes include the red blood cell membrane [39], platelet membrane [40], macrophage membrane [41], and stem cell membrane [42]. After stroke, peripheral macrophages are recruited to the central nervous system to participate in and regulate neuroinflammation. The interaction between pro-inflammatory factors and CD11b on macrophage membranes promotes macrophage infiltration [43,44]. Therefore, liposomes camouflaged by macrophage membranes can deliver drugs to ischemic areas of the brain, thereby achieving the goal of targeted therapy.

Due to the unique anatomical structure of the nasal cavity, drugs can be absorbed through the nose and transported to the brain [45]. Intranasal administration can bypass the blood–brain barrier and directly reach the central nervous system through the olfactory and trigeminal pathways [46,47]. Intranasal administration has advantages such as non-invasiveness, safety, and speed [48]. Nasal absorption avoids the liver first-pass effect and improves the bioavailability of nanomedicines in the brain [49]. It is currently a very promising route of administration for treating central nervous system diseases [50,51].

Network pharmacology is a powerful tool for analyzing the efficacy, bioavailability, and mechanism of action of drugs [52]. Network pharmacology is suitable for exploring the relationship between drugs and diseases. By constructing a “disease–gene–pathway– drug” network to analyze therapeutic targets and pathways, it has comprehensive and holistic characteristics [53].

Based on preliminary understanding, this study aimed to prepare a liposome coated with a macrophage membrane for the co-delivery of PNS and Rg3 to target the site of cerebral ischemia and improve therapeutic efficacy. First, the optimal prescription design of MM-Lip-Rg3/PNS was characterized in terms of its zeta potential, particle size, encapsulation efficiency, stability, and morphology. The mechanism of in vitro uptake of MM-Lip-Rg3/PNS was also investigated. Network pharmacology was used to predict key targets and pathways and facilitate in-depth research on therapeutic mechanisms. Finally, in vivo bio-experiments were used to further verify the anti-ischemic stroke effects of the liposomes. After the intranasal administration of MM-Lip-Rg3/PNS, we explored its effects on the survival rate, weight, neurological score, and anti-neuronal apoptotic ability of MCAO model rats to evaluate its ability to resist ischemic stroke. In conclusion, developing this biomimetic liposome is beneficial for the effective delivery of PNS and Rg3, providing valuable insights into the treatment of ischemic stroke.

## 2. Results and Discussion

### 2.1. Preparation and Optimization of Lip-Rg3/PNS

**Figure 1:**
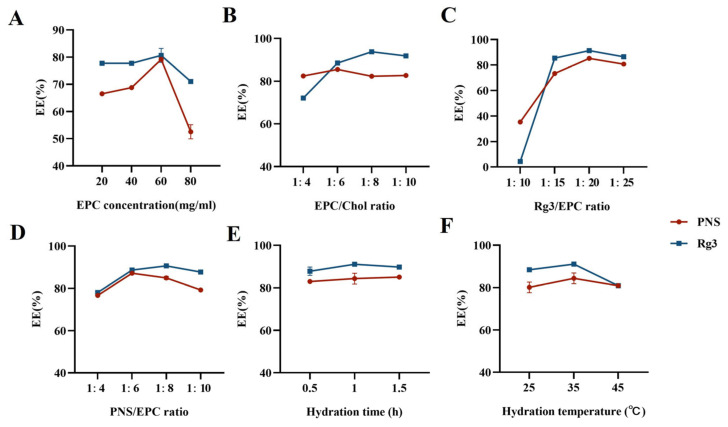
Single-factor investigation of Lip-Rg3/PNS. Influence of (**A**) EPC concentration, (**B**) EPC/Chol ratio, (**C**) Rg3/EPC ratio, (**D**) PNS/EPC ratio, (**E**) hydration time, and (**F**) hydration temperature on the encapsulation efficiency of Rg3 and PNS in Lip-Rg3/PNS.

Single-factor investigations were conducted using the encapsulation efficiencies of PNS and Rg3 as evaluation indicators (Figure 1). The influence of the EPC/Chol ratio, hydration temperature, and hydration time on the encapsulation efficiency (EE)% of PNS and Rg3 was relatively small. The optimal levels were selected, with an EPC/Chol ratio of 1:8, a hydration temperature of 35 °C, and a hydration time of 1 h. The EPC concentration, Rg3/EPC ratio, and PNS/EPC ratio were the three factors that significantly affected the EE% of PNS and Rg3. Therefore, these three factors were further investigated as experimental response surface factors.

The Box Behnken Design was used to optimize the formulation of the liposome by Design Expert.V8.0.6.1 (Stat-Ease, Minneapolis, MN, USA) [54], and a total of 17 experiments were performed to study the effect of three independent variables on the EE% of Rg3 and PNS. The experimental results are summarized in Table 1. Design Expert 8.0 software was used to perform regression model equation fitting on the obtained experimental data, and the regression simulation equations between the EE% of PNS (Y1) and the EE% of Rg3 (Y2) on three influencing factors (EPC concentration (A), PNS/EPC ratio (B), and Rg3/EPC ratio (C)) were obtained as follows:

$$\begin{array}{ll} Y_{1}\;= & +85.73-0.63\times A+0.48\times B+0.91\times C-0.72\times A\times B+0.35\times A\times C\\ & -0.055\times B\times C-2.64\times A^{2}-3.55\times B^{2}-1.89\times C^{2} \end{array}$$

$$\begin{array}{ll} Y_{2}\;= & +90.29-4.00\times A+1.01\times B+6.78\times C+1.85\times A\times B+2.36\times A\times C\\ & -0.27\times B\times C-2.59\times A^{2}-1.95\times B^{2}-3.42\times C^{2} \end{array}$$

The coefficients of determination (R2) of the above regression equations were 0.9790 and 0.9762, respectively, indicating the good fit of the equation [55]. An analysis of variance (ANOVA) was conducted on the regression model to investigate the degree of influence of various factors on EE%. The ANOVA results are shown in Table 2 and Table 3. The response of the EE% of PNS showed that a *p*-value < 0.0001 indicated a highly significant model, whereas an F-value of 0.4455 > 0.05 indicated that the error was not significant. The EPC concentration and Rg3/EPC ratio in the first-order term, as well as the three factors in the quadratic term, significantly impacted the EE% of PNS (*p* < 0.05). The response of the EE% of Rg3 (*p* < 0.0001) indicated that the model was highly significant, whereas an F-value of 0.3372 > 0.05 indicated that the error was not significant. The EPC concentration and Rg3/EPC ratio in the first-order term, as well as the three factors in the quadratic term, significantly impacted the EE% of PNS. The interaction between EPC concentration and the Rg3/EPC ratio, as well as between EPC concentration and the PNS/EPC ratio, was significant (*p* < 0.05).

**Figure 2:**
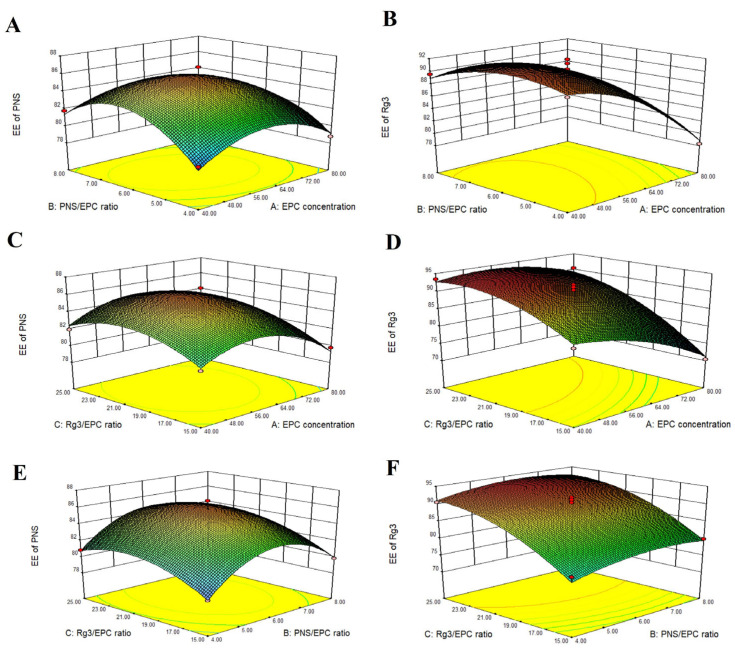
Three-dimensional response surface graphs showing the impact of three factors on encapsulation efficiency: The effect of the interaction between the EPC concentration and PNS/EPC ratio on the EE% of (**A**) PNS and (**B**) Rg3 in Lip-Rg3/PNS; the effect of interaction between EPC concentration and Rg3/EPC ratio on the EE% of (**C**) PNS and (**D**) Rg3 in Lip-Rg3/PNS; and the effect of interaction between PNS/EPC ratio and Rg3/EPC ratio on the EE% of (**E**) PNS and (**F**) Rg3 in Lip-Rg3/PNS.

The three-dimensional response surface graphs show the influence of the pairwise interactions of various factors on the EE% (Figure 2). The optimal prescription, based on the maximum EE% of PNS and Rg3, was predicted and validated. The results are summarized in Table 4. The optimal formulation for obtaining Lip-Rg3/PNS had an EPC concentration of 56 mg/mL, an Rg3/EPC ratio of 1:22, and a PNS/EPC ratio of 1:6.

### 2.2. Characterization of Liposomes

**Figure 3:**
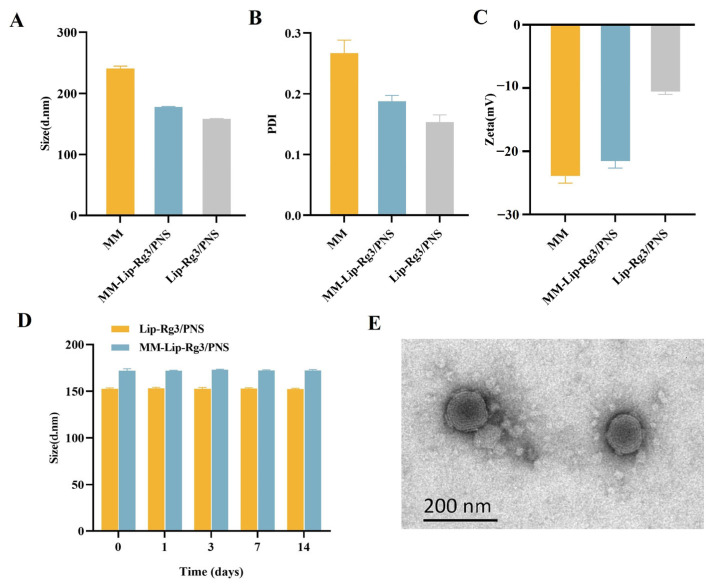
Characterization of MM, Lip-Rg3/PNS S, and MM-Lip-Rg3/PNS, including (**A**) particle size, (**B**) zeta potential, and (**C**) PDI. (**D**) Stability study of Lip-Rg3/PNS and MM-Lip-Rg3/PNS: particle size variation in different liposome formulations at room temperature (25 °C) for 14 days. (**E**) TEM image of MM-Lip-Rg3/PNS. Scale bar: 200 nm.

We used co-extrusion to obtain macrophage membrane-coated liposomes and evaluate the particle size, potential, and polydispersity index (PDI) of the obtained macrophage membrane microcapsules (MMs), Lip-Rg3/PNS, and MM-Lip-Rg3/PNS. The prepared MM particle size was 240.8 ± 3.7 nm, and the particle size of Lip-Rg3/PNS and MM-Lip-Rg3/PNS was 157.9 ± 0.66 nm and 177.7 ± 0.70 nm, respectively (Figure 3A). Compared to Lip-Rg3/PNS, the particle size of MM-Lip-Rg3/PNS increased by approximately 20 nm, indicating that the macrophage membrane was encapsulated on the liposome surface, leading to an increase in the particle size of the liposomes [56]. As shown in Figure 3B, the potential of MM-Lip-Rg3/PNS was −21.5 ± 1.11 mV, which was lower than the potential of Lip-Rg3/PNS (–10.6 ± 0.4 mV) and close to the potential of MM (–23.9 ± 1.2mV) [57]. The transmission electron microscopy (TEM) results showed that MM-Lip-Rg3/PNSs were spherical, with a membrane on the outer layer (Figure 3E), which is a core–shell structure, further indicating that the liposomes were successfully coated by the macrophage membrane [58]. The PDI values of both MM-Lip-Rg3/PNS and Lip-Rg3/PNS were less than 0.3, indicating good homogeneity (Figure 3C).

### 2.3. Encapsulation Efficiency and Drug Loading

The encapsulation efficiency (EE%) and drug loading (DL%) of Lip-Rg3/PNS and MM-Lip-Rg3/PNS are shown in Table 5. The EE% of PNS and Rg3 in Lip-Rg3/PNS was 83.33 ± 0.34% and 91.87 ± 1.30%, respectively. Both values were greater than 80%, indicating good encapsulation efficiency. The DL% of PNS and Rg3 in Lip-Rg3/PNS was 9.85 ± 0.04% and 2.83 ± 0.04%, respectively. After preparation as MM-Lip-Rg3/PNS, the EE% and DL% of the two drugs did not change significantly, implying that encapsulating the macrophage membrane did not lead to the leakage of liposome drugs.

### 2.4. Stability of Liposomes

Liposome stability is shown in Figure 3D. There was a small change in the particle sizes of Lip-Rg3/PNS and MM-Lip-Rg3/PNS, and it was maintained at approximately 150 nm and 170 nm, respectively, illustrating that Lip-Rg3/PNS and MM-Lip-Rg3/PNS can maintain storage stability for 14 days.

### 2.5. In Vitro Endocytosis Mechanism

**Figure 4:**
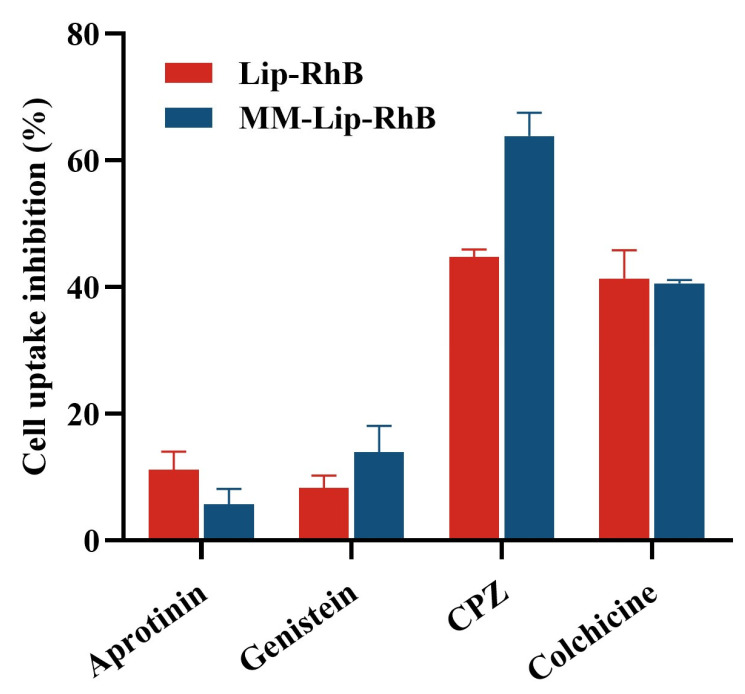
In vitro uptake mechanism of hCMEC/D3 cells for Lip-RhB and MM-Lip-RhB. Aprotinin, genistein, chlorpromazine (CPZ), and colchicine are four types of endocytosis inhibitors. Aprotinin competes with liposomes for the low-density lipoprotein receptor-related protein (LRP) receptors on endothelial membranes, genistein blocks caveolae-dependent endocytosis, CPZ inhibits clathrin-mediated endocytosis, and colchicine is a micropinocytosis inhibitor.

To clarify the cellular uptake mechanism of Lip-RhB and MM-Lip-RhB, hCMEC/D3 cells were treated with several endocytosis inhibitors, including chlorpromazine (CPZ), colchicine, aprotinin, and genistein, and cellular uptake was evaluated using flow cytometry. After treatment, the uptake of Lip-RhB and MM-Lip-RhB decreased by varying degrees (Figure 4). Aprotinin competes with liposomes for the low-density lipoprotein receptor-related protein (LRP) receptors on endothelial membranes, and genistein blocks caveolin-mediated endocytosis. These inhibitors had little effect on the uptake of Lip-RhB and MM-Lip-RhB. Colchicine is a micropinocytosis inhibitor that can reduce Lip-RhB and MM-Lip-RhB uptake in cells with similar degrees of inhibition and inhibition rates of 41.34% and 40.63%, respectively. CPZ inhibited clathrin-dependent endocytosis, resulting in a significant decrease in the uptake of both Lip-RhB and MM-Lip-RhB, corresponding to 44.8% and 63.87% uptake reductions, respectively, with more severe inhibition of MM-Lip-RhB. The above results indicated that the uptake mechanism of hCMEC/D3 cells for Lip-RhB and MM-Lip-RhB was not singular, and clathrin-dependent endocytosis and micropinocytosis were the main uptake pathways.

### 2.6. Network Pharmacology

#### 2.6.1. Prediction of Potential PNS Action Targets for Ischemic Stroke

**Figure 5:**
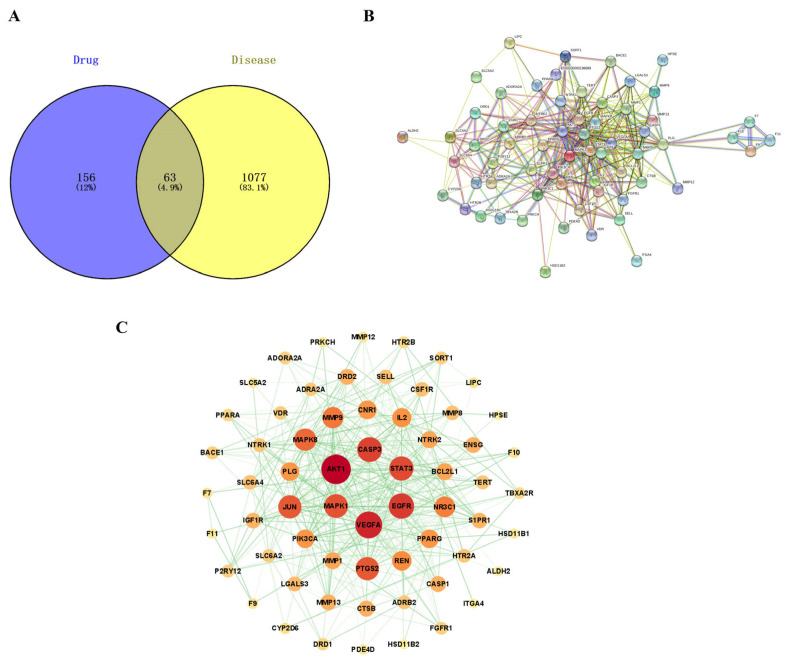
Prediction of interaction between drug active ingredients and disease targets, using network pharmacology. (**A**) Venn diagram of active ingredients and disease targets: drug represents PNS and Rg3, and disease represents ischemic stroke. The PPI network of potential targets against ischemic stroke. (**B**) The PPI network was obtained using STRING data. (**C**) The interaction network was obtained via processing with Cytoscape 3.9.1; the larger the node and darker the color, the greater the importance of the target.

To investigate the therapeutic mechanisms of PNS and Rg3 in ischemic stroke, we conducted a network pharmacology study to predict the key targets and main signaling pathways involved in the combined use of these two drugs in ischemic stroke. A total of 219 potential targets of PNS and Rg3 were predicted using the SwissTargetPrediction database. We integrated ischemic stroke targets predicted from GeneCards and the DisGeNET database and removed duplicates and low-correlation targets through median screening, resulting in a total of 1140 disease targets. The intersection of the PNS and Rg3 targets with disease targets resulted in 63 common targets (Figure 5A).

#### 2.6.2. PPI Network Construction

After analyzing the intersecting targets using the STRING database, a PPI network diagram was obtained. The diagram included 63 nodes and 412 edges (Figure 5B). The data file obtained from the STRING database was imported into Cytoscape 3.9.1 software to construct a target interaction network (Figure 5C). In this network, the larger the node and darker the color, the greater the degree value and importance of the target; and the thicker the line and darker the color, the stronger the interaction. The targets of the network center, including AKT1, VEGFA, EGFR, CASP3, STAT3, and MAPK1, had high degree values, indicating that they may be key targets in the regulation of ischemic stroke.

#### 2.6.3. GO and KEGG Analysis

**Figure 6:**
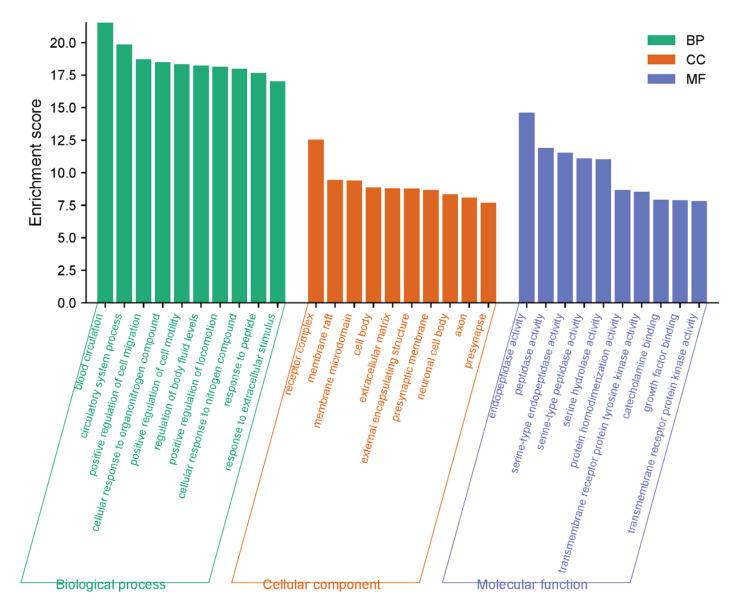
GO-enrichment histogram of potential therapeutic target genes, including biological processes, cellular components, and molecular functions.

The biological characteristics and related signaling pathways of PNS and Rg3 in ischemic stroke were analyzed using gene ontology (GO) enrichment and the Kyoto Encyclopedia of Genes and Genomes (KEGG). In biological processes (BPs), targets mainly involved blood circulation, circulatory system processes, the positive regulation of cell migration, the cellular response to organonitrogen compounds, and the positive regulation of cell motility. The cellular components (CCs) mainly involved the receptor complex, membrane raft, membrane microdomain, cell body, and extracellular matrix. The molecular functions (MFs) mainly involved endopeptidase activity, peptidase activity, serine-type endopeptidase activity, serine-type peptidase activity, and serine hydrolase activity (Figure 6).

**Figure 7:**
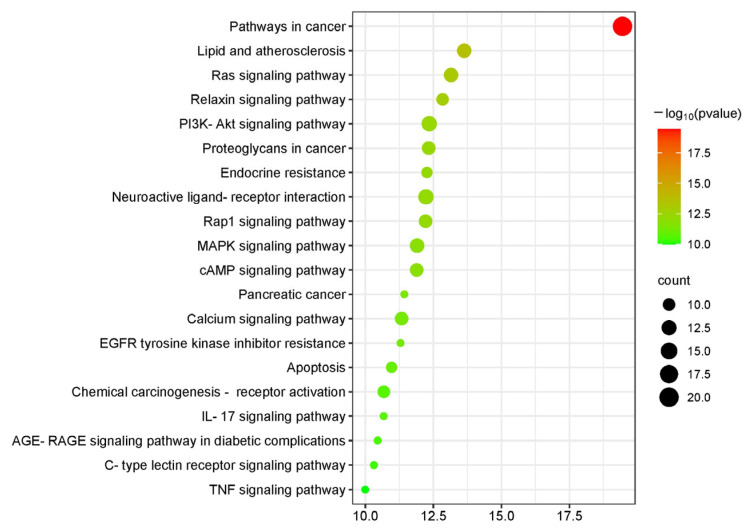
KEGG-enrichment bubble plot. The darker the color of the bubble, the higher the *p*-value; the larger the bubble, the higher the degree of enrichment.

The signal pathways obtained from the KEGG analysis were sorted by *p*-values, and the top 20 related signaling pathways were plotted as a bubble plot for visual analysis (Figure 7). The color depth of the bubbles was positively correlated with the *p*-value, and the size of the bubbles was positively correlated with the number of enriched targets in the pathway.

According to the KEGG analysis, the pathways related to the treatment of ischemic stroke mainly involved the Ras signaling pathway, PI3K-Akt signaling pathway, neuroactive ligand–receptor interaction, Rap1 signaling pathway, MAPK signaling pathway, cAMP signaling pathway, apoptosis, IL-17 signaling pathway, C-type lectin receptor signaling pathway, and TNF signaling pathway. Numerous studies have shown that the PI3K/Akt signaling pathway can inhibit neuronal apoptosis and promote neuronal growth [59]. Akt is a key protein in the PI3K/Akt pathway, and phosphorylated Akt further activates downstream proteins that participate in cell growth and development processes [60]. Following a stroke, the PI3K Akt pathway maintains brain homeostasis by regulating various target proteins; is an important pathway for protecting brain nerves [61]. The MAPK signaling pathway plays an important regulatory role in growth, metabolism, apoptosis, and oxidative stress [62]. The MAPK signaling pathway mainly includes the p38 MAPK, JNK MAPK, ERK1/2 MAPK, and ERK5 MAPK pathways, all of which are activated in ischemic stroke [63,64]. The activated cAMP signaling pathway can regulate microglia and astrocytes to reduce inflammation, promote angiogenesis, and restore nerve cell function, thus reducing stroke damage [65,66]. The calcium signaling pathway is closely related to thrombosis and cell necrosis [67]. Rap is a member of the Ras superfamily, and the activated Rap signaling pathway can reduce brain edema and protect the damaged blood–brain barrier [68]. IL-17 signaling pathway and TNF signaling pathway are primarily involved in immune and inflammatory responses [69]. In summary, the combined application of PNS and Rg3 to treat ischemic stroke mainly involves inflammation, cell apoptosis, angiogenesis, and neuronal protection. The results of network pharmacology experiments indicated that the combination of PNS and Rg3 may play an important role in the treatment of ischemic stroke. This study provides a reference for the systematic study of PNS and Rg3 in the treatment of ischemic stroke.

#### 2.6.4. The “Component–Gene–Pathway” Network

**Figure 8:**
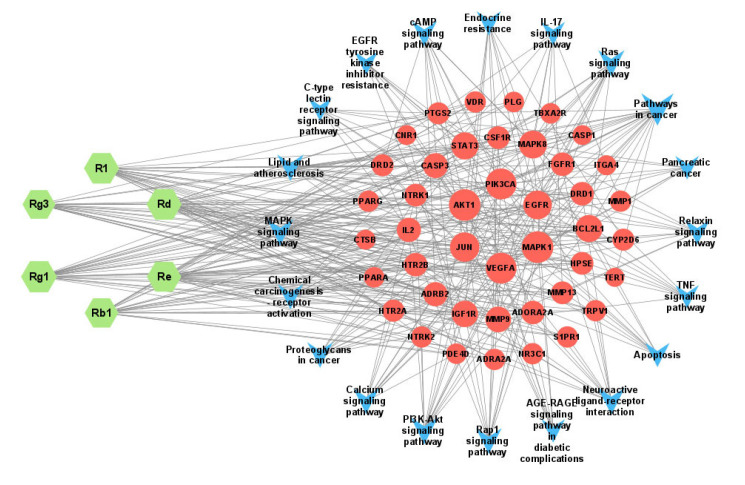
“Component–gene–pathway” network. The blue arrow represents the signaling pathway; the green hexagon represents the component; the red circle represents the target; and the edges represent interactions between compounds, target proteins, and signaling pathways. The larger the node, the greater the degree value.

To accurately and comprehensively illustrate the potential mechanisms of the action of PNS and Rg3 in treating ischemic stroke, we constructed a “component–gene–pathway” network diagram. The diagram included the main active ingredients, the top 20 key pathways, and their corresponding targets. The resulting network graph contained 69 nodes and 350 edges (Figure 8). The network graph indicated that Rg1 was the most important component among the six main components. It had the highest degree value, the most connected edges, and the most corresponding targets. Therefore, Rg1 plays a crucial role in this network, suggesting that ginsenoside Rg1 is a key component in the treatment of ischemic stroke. When the degree was greater than or equal to 15, the targets screened for PNS and Rg3 to treat ischemic stroke through the first 20 pathways were MAPK1, AKT1, PIK3CA, VEGF, JUN, and EGFR. These targets have degree values of 19, 19, 18, 18, 16, and 15, respectively, and may be considered core targets. These results indicate that various biological pathways, such as the Rap1, MAPK, cAMP, IL-17, and TNF signaling pathways, are involved in the inflammatory response, oxidative stress, promotion of angiogenesis, and alleviation of neuronal damage to effectively treat ischemic stroke. Thus, it can be demonstrated that PNS and Rg3 exhibit anti-ischemic stroke effects with the characteristics of “multiple components, multiple targets, and multiple pathways.”

### 2.7. The Therapeutic Effect of Anti-Ischemic Stroke

**Figure 9:**
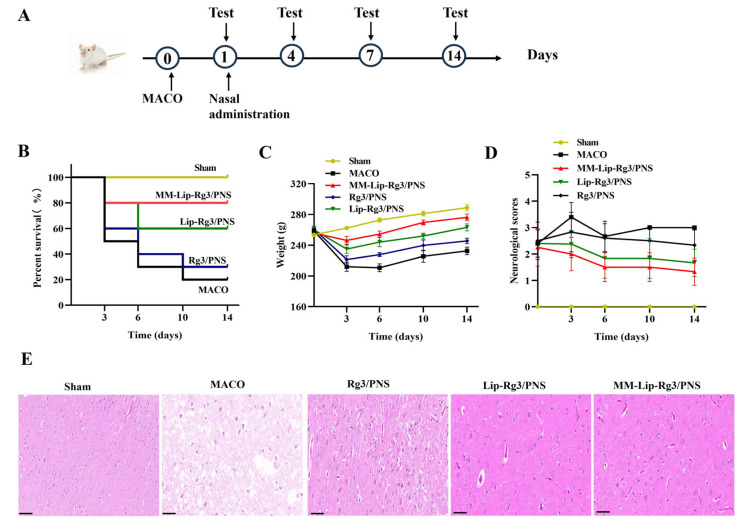
Therapeutic effects of different formulations on ischemic stroke. (**A**) Experimental scheme, (**B**) survival rate, (**C**) weight changes, (**D**) neurological score, and (**E**) hematoxylin and eosin staining of the brains from each group (scale bars: 50 μm). Different formulations administered via the intranasal pathway can promote brain transport.

We evaluated the therapeutic effects of the different formulations by using MCAO model rats; the specific treatment process is shown in Figure 9A. Rats in the MCAO group that received no medication died continuously within 14 days, resulting in a survival rate of only 20%. However, the survival rates of all three treatment groups improved, with the MM-Lip-Rg3/PNS group showing no deaths after three days and reaching a survival rate of 80%. Sham rats continued to gain weight in 14 days (Figure 9B). MCAO rats experienced delayed movement and mental exhaustion after ischemia, leading to continuous weight loss within six days, with a rapid weight loss in the first three days. After six days, the MCAO rats could consume a small amount of food, resulting in a slight increase in weight. Treatment with Rg3/PNS, Lip-Rg3/PNS, and MM-Lip-Rg3/PNS alleviated the symptoms of cerebral ischemia in rats and slowed the rate of weight loss. MM-Lip-Rg3/PNS was effective, significantly inhibiting weight loss and resulting in a faster recovery than the other two treatments. After 6 days of treatment, the weight of the rats was higher than that before surgery and continued to increase (Figure 9C). The neurological scoring results showed that the MCAO group had higher scores, which did not decrease after 14 days. The Rg3/PNS group showed a slight decrease in score compared with the MCAO group, but the rats remained above 2 points and were unable to walk normally. The Lip-Rg3/PNS and MM-Lip-Rg3/PNS groups’ scores were significantly reduced, especially the MM-Lip-Rg3/PNS group. After 14 days of administration, the rats were able to move freely and eat normally, exhibiting behavior similar to that of the sham group (Figure 9D). These results suggest that PNS and Rg3 have therapeutic effects on cerebral ischemia, mainly MM-Lip-Rg3/PNS, and can effectively restore neural function in MCAO rats, allowing for a good survival rate and improved quality of life.

### 2.8. H&E Staining of Brain Tissue

H&E staining was used to observe histological changes in the brain to verify the protective effects of the different formulations on the brain after ischemic stroke (Figure 9E). The neuronal cells in the sham group showed an intact morphology, an orderly arrangement, abundant cytoplasm, and clearly visible nuclei and nuclear membranes [70]. However, the MCAO model group showed a significant decrease in the number of cells, nuclear membrane rupture, irregular cell morphology, structural changes, cytoplasmic loss, and the appearance of vacuoles. Treatment with Rg3/PNS, Lip-Rg3/PNS, and MM-Lip-Rg3/PNS reduced brain tissue damage. The MM-Lip-Rg3/PNS group showed an increase in the number of normal cells, a decrease in cell necrosis, an increase in cytoplasm, and an improvement in cell structure, which approached normal brain morphology. These results suggest that macrophage biomimetic liposomes have a significant protective effect on the ischemic brain and may treat brain damage.

### 2.9. MM-Lip-Rg3/PNSs Reduced Neuronal Apoptosis Caused by Ischemia

**Figure 10:**
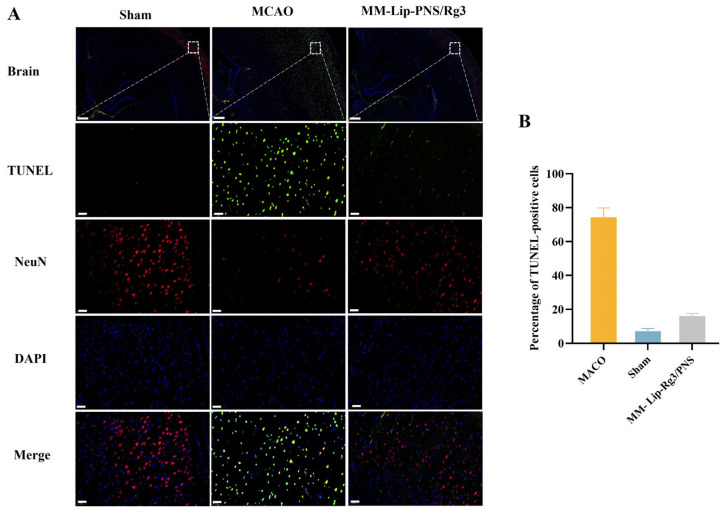
The effect of MM-Lip-Rg3/PNSs on brain apoptosis in rats after MCAO. (**A**) Immunofluorescence staining of TUNEL and NeuN in the ischemic hemisphere. TUNEL-positive cells, green; NeuN, red; DAPI, blue (scale bars: 500/50 μm). (**B**) The percentage of TUNEL-positive cells in each formulation group. MM-Lip-Rg3/PNS can reduce the number of apoptotic neurons.

To verify the anti-apoptotic ability of MM-Lip-Rg3/PNSs against cerebral ischemic injury, TUNEL staining and NeuN staining were performed. NeuN is a specific protein expressed in neuronal cells [71], and the TUNEL assay can be used to label apoptotic cells [72]. There were a considerable number of NeuN cells, but almost no TUNEL cells were observed in the cerebral cortex of the Sham group. The NeuN cells in the MCAO group were few, while many TUNEL cells were present, indicating severe neuronal necrosis in the MCAO model rats. The number of NeuN cells in the MM-Lip-Rg3/PNS group increased and the number of TUNEL cells decreased compared to that in the MCAO group, suggesting that the MM-Lip-Rg3/PNSs reduced cell apoptosis and improved the neuronal count (Figure 10). These results suggest that MM-Lip-Rg3/PNSs can alleviate neuronal damage caused by cerebral ischemia and protect the brain tissue.

## 3. Materials and Methods

### 3.1. Materials

Egg yolk phosphatidylcholine was supplied by Lipoid (Cologne, Germany). PNS was purchased from Dilger Co., Ltd. (Nanjing, China). Rg3 was supplied by Ytbiopharm Inc. (Changchun, China). Methanol, acetonitrile, and chloroform were purchased from YUHE New Mat Co., Ltd. (Yantai, China).

### 3.2. Cell Culture

The mouse monocyte–macrophage cell line RAW264.7 was purchased from Pricella Bioscience Co., Ltd. (Wuhan, China). Cells were cultured in DMEM supplemented with 10% fetal bovine serum (FBS) at 37 °C, under 5% CO_2_. The human cerebral microvessel endothelial cell line (hCMEC/D3) was purchased from Deltabio Co., Ltd. (Chengdu, China). Cells were cultured in an endothelial cell medium supplemented with 5% FBS, 1% cell growth factors, and 1% penicillin/streptomycin (ScienCell Co., Ltd. Beijing, China) at 37 °C, under 5% CO_2_.

### 3.3. Preparation Method of Lip-Rg3/PNS

Lip-Rg3/PNS was prepared using a thin-film dispersion method [73]. Firstly, egg yolk lecithin (EPC), Rg3, and cholesterol (Chol) were dissolved in a chloroform–methanol (2:3) solution and then spin-dried at 37 °C to form a thin film. The thin film was hydrated with PNS aqueous solution to obtain Lip-Rg3/PNS.

### 3.4. Determination of Encapsulation Efficiency and Drug Loading

PNS is water-soluble; therefore, ultrafiltration centrifugation was used to separate free drugs. Lip-Rg3/PNS was added to the inner tube of 100 K Amicon^®^ Ultra-4 central filters and centrifuged at 4000 rpm for 20 min. The content of free PNS in the filtrate was then measured. Rg3 is insoluble in water and has high non-polarity; therefore, low-speed centrifugation was used to separate the free drugs. Lip-Rg3/PNS was centrifuged at 3000 rpm for 10 min to determine the free Rg3 content in the supernatant. The equations for EE% and DL% were as follows:

$$\mathrm{EE}\%=\frac{\mathrm{Amount}\;\mathrm{of}\;\mathrm{encapsulated}\;\mathrm{drug}}{\mathrm{Amount}\;\mathrm{of}\;\mathrm{total}\;\mathrm{drug}}\times 100\%$$

$$\mathrm{DL}\%=\frac{\mathrm{Weight}\;\mathrm{of}\;\mathrm{encapsulated}\;\mathrm{drug}}{\mathrm{Weight}\;\mathrm{of}\;\mathrm{Liposome}}\times 100\%$$

### 3.5. Optimization Design of Lip-Rg3/PNS

#### 3.5.1. Single-Factor Experiment

We evaluated the EPC concentration, EPC/Chol ratio, Rg3/EPC ratio, PNS/EPC ratio, hydration temperature, and hydration time to identify factors that significantly impacted liposome preparation.

#### 3.5.2. Box–Behnken Design

Based on single-factor experiments, the EPC concentration, Rg3/EPC ratio, and PNS/EPC ratio were selected as independent variables. The encapsulation efficiencies of PNS and Rg3 were used as dependent variables to conduct response surface experiments and optimize the preparation process [74]. The experimental factors and response surface-design levels are listed in Table 6.

### 3.6. Preparation of MM-Lip-Rg3/PNS

An ultrasonic cell breaker was used to extract macrophage membranes (MMs) and mix them with liposomes. After repeated squeezing with an extruder, biomimetic liposomes coated with macrophage membranes (MM-Lip-Rg3/PNSs) were formed.

### 3.7. Characterization of Liposomes

Different liposomes formulations were diluted 100 times with distilled water, and particle size, polydispersity index (PDI), and zeta potential were measured using a dynamic light scattering (Nano-ZS90, Malvern Panalytics, Malvern, UK). MM-Lip-Rg3/PNS morphology was observed using transmission electron microscopy (TEM) (Hitachi, Tokyo, Japan).

### 3.8. Stability of Liposomes

To study the stability of liposomes, Lip-Rg3/PNS and MM-Lip-Rg3/PNS were stored at room temperature for 14 days, and the particle size was measured at 0, 1, 3, 7, and 14 days to record the changes in particle size.

### 3.9. In Vitro Endocytosis Mechanism

Aprotinin, genistein, colchicine, and chlorpromazine endocytosis inhibitors were used to investigate the mechanism underlying hCMEC/D3 the cell uptake of ordinary and MM-coated liposomes. Briefly, 3 × 105 hCMEC/D3 cells were seeded in a 6-well plate. Aprotinin (10 μM), genistein (20 μM), colchicine (20 μM), and chlorpromazine (20 μM) were each added separately and incubated with hCMEC/D3 cells for 1 h. The culture medium was discarded, and normal culture media containing Lip-RhB and MM-Lip-RhB (RhB concentration in each well was 10.0 μg/mL) was added, followed by incubation for 3 h. The cells were collected, and their fluorescence intensity was measured using flow cytometry.

### 3.10. Network Pharmacology

#### 3.10.1. Collection of Action Targets of PNS Active Ingredients

Five main active ingredients in PNS, namely ginsenoside Rb1, ginsenoside Rg1, ginsenoside Rd, notoginsenoside R1, and ginsenoside Re, as well as ginsenoside Rg3, were selected as candidate components for network pharmacology analysis. The SMILES number of the compound was obtained from the PubChem database (http://pubchem.ncbi.nlm.nih.gov/) and imported into the Swiss Target Prediction database (http://www.swisstargetprediction.ch/) to predict the target genes of the five components mentioned above.

#### 3.10.2. Collection of Target Genes Related to Ischemic Stroke

Target genes related to ischemic stroke were screened in the GeneCards database (https://www.genecards.org/) and the DisGeNET database (https://www.disgenet.org/).

#### 3.10.3. Prediction of Potential Targets of Drug in Ischemic Stroke

The interacting targets were obtained by introducing the targets of active ingredients and ischemic stroke targets into venny 2.1.0 (https://bioinfogp.cnb.csic.es/tools/venny/).

#### 3.10.4. Protein–Protein Interaction (PPI) Network Construction

The obtained interacting targets were uploaded to the STRING database (https://string-db.org/), and “Homo sapiens” was selected as the research species to construct a PPI network plot. The PPI file obtained from STRING was input into Cytoscape 3.9.1 software for analysis to obtain a more intuitive network plot that reflects the interactions between proteins.

#### 3.10.5. Enrichment Analysis

The common targets of components and diseases were input into the Metascape database (https://metascape.org/) for GO enrichment and KEGG pathway analysis, and the data were visualized using a bioinformatics website (https://www.bioinformatics.com.cn/) for processing. Finally, we utilized the Cytoscape 3.9.1 software to create a “component–gene–pathway” network.

### 3.11. Establish a Middle Cerebral Art Occlusion Model

Sprague Dawley (SD) rats were selected as the experimental animals and purchased from Changchun Yisi Experimental Animal Technology Co., Ltd. (Changchun, China). All experimental procedures were approved by the Experimental Animal Ethics Committee and strictly followed the requirements.

The MCAO model was established using the suture method. Rats were anesthetized with 1% sodium pentobarbital, and the common carotid artery was dissected. After cutting a small incision in the internal carotid artery, the suture was inserted, and the wound was sutured.

### 3.12. Evaluate the Therapeutic Effects of Different Liposome Formulations

Treatment of rats after 1 day after MCAO: The MCAO rats were treated with saline, free PNS, Rg3, Lip-Rg3/PNS, and MM-Lip -Rg3/PNS. The rats were divided into five groups as follows: MCAO (only physiological saline), Rg3/PNS (free PNS and Rg3), Lip-Rg3/PNS (liposomes loaded with Rg3 and PNS), MM-Lip-Rg3/PNS (MM-Lipo loaded with Rg3 and PNS), and sham (sham-operated control). Each group included ten rats.

Body weight and postoperative neurobehavioral scores were collected for each group on days 0, 3, 6, 10, and 14 of treatment. Neurological scoring was performed using the Longa 5-point scoring standard, with each rat blindly tested three times by two examiners.

### 3.13. H&E Staining of the Brain in Different Treatment Groups

After sacrificing the rats in the different treatment groups, brain tissues were removed, fixed with paraformaldehyde, soaked in xylene, and sliced. The slices were then baked, deparaffinized, rehydrated, stained with hematoxylin blue, sealed, and dried before observation under an electron microscope.

### 3.14. TUNEL and NeuN Staining of Brain Tissue

MCAO rats were given MMs via intranasal administration for 14 days. Rat brains were removed after sacrificing the rats. Brain tissue was sliced into paraffin sections, dewaxed, repaired, membrane-ruptured, and incubated with TdT and dUTP (Roche Co., Ltd., Shanghai, China). The primary (NeuN) and secondary antibodies were added separately (Servicebio Co., Ltd., Wuhan, China). Finally, DAPI (Beyotime Co., Ltd., Shanghai, China) was added in a dropwise manner to re-stain the cell nucleus. After sealing, a fluorescence microscope (Nikon, Tokyo, Japan) was used to observe and capture images, and ImageJ software (https://imagej.net/ij/, National Institutes of Health, Bethesda, MD, USA) was used for counting.

### 3.15. Statistical Analysis

All measurements were independently repeated at least three times. All data were mean ± standard deviation (SD). One-way analysis of variance was used for statistical analysis when the *p*-value was <0.05.

## 4. Conclusions

In this study, Lip-Rg3/PNSs were prepared using the film dispersion method, and the optimal formulation conditions were determined by response surface methodology. The results of measuring the encapsulation efficiencies of PNS and Rg3 in Lip-Rg3/PNS were consistent with the predicted values. MM-Lip-Rg3/PNSs were obtained via the co-extrusion of Lip-Rg3/PNS and the macrophage membrane, and transmission electron microscopy showed that the MM-Lip-Rg3/PNSs were spherical, with a membrane coating on the outer layer. In addition, the MM-Lip-Rg3/PNSs exhibited good stability at room temperature and 4 °C. The cellular uptake mechanism of MM-Lip-Rg3/PNSs involves clathrin-dependent endocytosis and micropinocytosis. Network pharmacology was used to systematically explore the therapeutic mechanism of MM-Lip-Rg3/PNS against ischemic stroke. The analysis results indicated that MM-Lip-Rg3/PNS can treat ischemic stroke by reducing cell apoptosis, inflammatory response, and oxidative stress through multiple targets (including AKT1, VEGFA, EGFR, CASP3, STAT3, and MAPK1) and multiple pathways (including PI3K/Rap1/cAMP/IL-17/TNF signaling pathway), as confirmed through in vivo animal experiments. After intranasal administration of MM-Lip-Rg3/PNS, the survival rate of MCAO rats increased, and neurological function improved. The H&E staining showed that the MM-Lip-Rg3/PNSs improved the histological morphology of the brain and reduced inflammation, and TUNEL immunofluorescence showed that the MM-Lip-Rg3/PNSs inhibited neuronal apoptosis. The in vivo biological experiments showed that both free PNS/Rg3 and MM-Lip-Rg3/PNSs have the effect of reducing cerebral ischemic injury, especially MM-Lip-Rg3/PNSs. Based on these results, we speculate that the development of MM-Lip-Rg3/PNSs may provide new and valuable ideas for treating ischemic stroke.

## Figures and Tables

**Table 1:** Design experimental results of Box–Behnken response surface method.

Run	Factor			Response: EE%	
	A	B	C	PNS	Rg3
1	40.00	1:4	1:20	79.34	90.26
2	80.00	1:4	1:20	78.75	78.28
3	40.00	1:8	1:20	81.77	89.54
4	80.00	1:8	1:20	78.31	84.94
5	40.00	1:6	1:15	80.95	82.83
6	80.00	1:6	1:15	79.75	70.41
7	40.00	1:6	1:25	81.95	93.45
8	80.00	1:6	1:25	82.14	90.46
9	60.00	1:4	1:15	78.8	78.25
10	60.00	1:8	1:15	79.84	79.84
11	60.00	1:4	1:25	80.85	90.55
12	60.00	1:8	1:25	81.67	91.07
13	60.00	1:6	1:20	86.74	91.87
14	60.00	1:6	1:20	85.71	89.59
15	60.00	1:6	1:20	85.51	91.22
16	60.00	1:6	1:20	85.15	88.43
17	60.00	1:6	1:20	85.56	90.36

Notes: (A) EPC concentration, (B) PNS/EPC ratio, and (C) Rg3/EPC ratio.

**Table 2:** Variance analysis of EE% (PNS) regression model (ANOVA).

Parameters	Sum of Squares	Degree of Freedom	Mean Square	F Value	*p*-Value
Model	122.27	9	13.59	36.28	<0.0001
A	3.20	1	3.20	8.55	0.0222
B	1.85	1	1.85	4.95	0.0615
C	6.61	1	6.61	17.64	0.0040
AB	2.06	1	2.06	5.50	0.0515
AC	0.48	1	0.48	1.29	0.2935
BC	0.012	1	0.012	0.032	0.8624
A2	29.39	1	29.39	78.48	<0.0001
B2	53.05	1	53.05	141.65	<0.0001
C2	15.11	1	15.11	40.35	0.0004
Residual	2.62	7	0.37		
Lack of fit	1.19	3	0.40	1.10	0.4450
Pure error	1.43	4	0.36		
Cor total	124.89	16			

Notes: (A) EPC concentration, (B) PNS/EPC ratio, and (C) Rg3/EPC ratio.

**Table 3:** Variance analysis of EE% (Rg3) regression model (ANOVA).

Parameters	Sum of Squares	Degree of Freedom	Mean Square	F Value	*p*-Value
Model	642.98	9	71.44	31.87	<0.0001
A	127.92	1	127.92	57.06	0.0001
B	8.10	1	8.10	3.61	0.0991
C	367.20	1	367.20	163.80	<0.0001
AB	13.62	1	13.62	6.07	0.0432
AC	22.23	1	22.23	9.92	0.0162
BC	0.29	1	0.29	0.13	0.7314
A2	28.23	1	28.23	12.59	0.0094
B2	16.00	1	16.00	7.14	0.0319
C2	49.16	1	49.16	21.93	0.0023
Residual	15.69	7	2.24		
Lack of fit	8.38	3	2.79	1.53	0.3372
Pure error	7.32	4	1.83		
Cor total	658.67	16			

Notes: (A) EPC concentration, (B) PNS/EPC ratio, and (C) Rg3/EPC ratio.

**Table 4:** Predicted and measured values of response surface optimization prescriptions (n = 3).

Values	Predicted Value	Experimental Values
EE% (PNS)	85.75%	83.33 ± 0.34%
EE% (Rg3)	93.26%	91.87 ± 1.30%

**Table 5:** The EE and DL of different liposome formulations (n = 3).

Formulation	EE (%)		DL (%)	
	PNS	Rg3	PNS	Rg3
Lip-Rg3/PNS	83.33 ± 0.34%	91.87 ± 1.30%	9.85 ± 0.04%	2.83 ± 0.04%
MM-Lip-Rg3/PNS	81.41 ± 2.25%	93.81 ± 1.56%	9.33 ± 0.24%	3.43 ± 0.05%

**Table 6:** The factors and levels of response surface design.

Factors	Levels		
	−1	0	1
EPC concentration (mg/mL)	40	60	80
PNS/EPC ratio	1:4	1:6	1:8
Rg3/EPC ratio	1:15	1:20	1:25

## Data Availability

All data supporting the findings of this study are presented graphically or in tables in this manuscript.
